# Polydatin Prevents UVA-Induced Damage in Human Dermal Fibroblasts by Maintaining Mitochondrial Integrity

**DOI:** 10.3390/cells14211702

**Published:** 2025-10-30

**Authors:** Benedetta Niccolini, Alessia Riente, Duaa Hatem, Patrizia Bottoni, Michela Pizzoferrato, Giuseppe Tringali, Elisabetta Tabolacci, Giuseppe Maulucci, Stefano Marini, Chiara Ciaccio, Maria Elisabetta Clementi

**Affiliations:** 1 UOC Genetica Medica, Fondazione Policlinico Universitario A. Gemelli IRCCS, 00168 Rome, Italy; benedetta.niccolini@unicatt.it (B.N.); elisabetta.tabolacci@unicatt.it (E.T.); 2 Dipartimento di Sanità Pubblica e Scienze della Vita, Sezione di Medicina Genomica, Università Cattolica del Sacro Cuore, 00168 Rome, Italy; 3 Fondazione Policlinico Universitario A. Gemelli IRCSS, 00168 Rome, Italy; alessia.riente@unicatt.it (A.R.); duaa.hatem@unicatt.it (D.H.); patrizia.bottoni@unicatt.it (P.B.); michela.pizzoferrato@unicatt.it (M.P.); giuseppe.tringali@unicatt.it (G.T.); giuseppe.maulucci@unicatt.it (G.M.); 4 Dipartimento di Neuroscienze, Sezione di Fisica, Università Cattolica del Sacro Cuore, 00168 Rome, Italy; 5 Department of Translational Medicine and Surgery, Università Cattolica del Sacro Cuore, 00168 Rome, Italy; 6 Pharmacology Section, Department of Health Care Surveillance and Bioethics, Università Cattolica del Sacro Cuore, 00168 Rome, Italy; 7 Department of Clinical Sciences and Translational Medicine, University of Rome ‘Tor Vergata’, Via Montpellier 1, 00133 Rome, Italy; stefano.marini@uniroma2.it (S.M.); chiara.ciaccio@uniroma2.it (C.C.); 8 Istituto di Scienze e Tecnologie Chimiche “Giulio Natta” SCITEC-CNR, 00168 Rome, Italy

**Keywords:** polydatin, antioxidant compounds, UVA-induced damage, oxidative stress, mitochondrial function

## Abstract

UVA radiation induces oxidative stress, mitochondrial dysfunction, and cell death in human dermal fibroblasts, contributing to skin aging and damage. In this study, we investigated the protective effects of polydatin, a natural polyphenol, against UVA-induced cell damage. Our results show that polydatin preserves cell viability and reduces intracellular reactive oxygen species (ROS) levels after UVA exposure. In addition, polydatin maintains mitochondrial integrity by preserving mitochondrial membrane potential and improving mitochondrial respiration. From a molecular perspective, polydatin regulates the expression of Nrf2, a key regulator of the cellular antioxidant response, thereby promoting cellular defense mechanisms. Additionally, polydatin attenuates UVA-induced mitochondrial fission, supporting a balanced mitochondrial dynamic profile. These results suggest that polydatin exerts a protective effect on UVA-irradiated fibroblasts, highlighting its potential for cosmetic and dermatological applications aimed at preventing photoaging and oxidative skin damage.

## 1. Introduction

**Figure 1:**
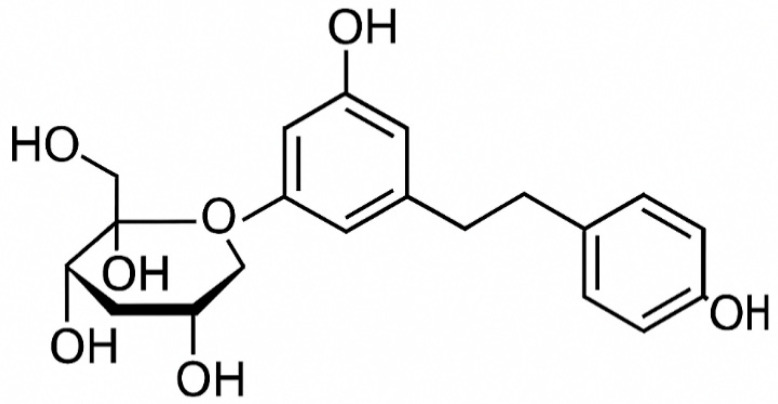
Chemical structure of polydatin.

Polydatin (piceid; 3,4′,5-trihydroxystilbene-3-β-D-glucoside) is a glycosylated stilbenoid derivative of resveratrol (see Figure 1), naturally found in Polygonum cuspidatum, grapes, peanuts, and red wine. The presence of a glucose moiety enhances its chemical stability and oral bioavailability compared to resveratrol, resulting in improved cellular uptake, enhanced metabolic stability, and reduced susceptibility to enzymatic oxidation [1,2,3]. A vast body of preclinical research has documented the multifaceted bioactivities of the trans-isomer of polydatin: it exerts potent antioxidant and anti-inflammatory effects, modulates apoptotic and fibrotic pathways, and provides protection across diverse tissues including the heart, liver, lungs, and brain [4,5,6]. Polydatin has been approved by the U.S. Food and Drug Administration (FDA) for use in both adult and pediatric populations and has been evaluated in clinical trials for a range of disorders [6,7,8].

Recent evidence has further clarified the role of polydatin as a context-dependent modulator of redox homeostasis. Although generally recognized for its antioxidant activity in normal cells, polydatin can exert a pro-oxidant effect under specific pathological conditions such as cancer. Using cell-based biochemical assays, Cimmino et al. demonstrated that cytotoxic concentrations of polydatin (100–200 µM) induce a marked increase in reactive oxygen species (ROS) production, accompanied by depletion of reduced glutathione (GSH) and accumulation of lipid peroxidation products such as malondialdehyde (MDA) in human osteosarcoma cell lines (SAOS-2 and U2OS) [9].

This dose-dependent shift toward oxidative stress suggests that, at high concentrations, polydatin’s pro-oxidant activity contributes to cancer cell damage and apoptosis, supporting its dual redox-modulating role depending on both the cellular context and the dosage.

Despite this wealth of data, relatively little is known about the potential role of radiation in skin protection, particularly in the context of photoinduced oxidative injury. UVA radiation (320–400 nm) penetrates deeply into the dermis, generating ROSs that provoke mitochondrial dysfunction, DNA lesions, activation of matrix metalloproteinases, and ultimately photoaging and cellular senescence. Among these events, mitochondrial damage plays a central role: mitochondria are not only major sources of ROS upon UVA exposure, but also key regulators of apoptosis, bioenergetics, and redox homeostasis [7,8]. While UVB radiation (280–320 nm) is mostly absorbed by the epidermis, its ability to reach the dermis is very limited compared to UVA, which is therefore the main driver of the dermal responses observed.

Excessive mitochondrial ROS generation under UVA stress disrupts mitochondrial dynamics, particularly the delicate balance between fission and fusion [10].

Mitochondrial fusion normally promotes the exchange of mitochondrial DNA, proteins, and metabolites, thereby sustaining bioenergetic efficiency and integrity against oxidative damage [11,12]. Conversely, mitochondrial fission is essential for quality control, allowing segregation of damaged organelles for mitophagy [11,12]. However, UVA-induced oxidative stress skews this equilibrium toward excessive fission and impaired fusion, resulting in fragmented, dysfunctional mitochondria. This abnormal remodeling reduces ATP production and exacerbates ROS accumulation, fueling a vicious cycle of mitochondrial injury [9,13,14].

A crucial marker of mitochondrial integrity in this context is the mitochondrial membrane potential (ΔΨm). Loss of ΔΨm reflects depolarization of the inner mitochondrial membrane, which compromises ATP synthesis and facilitates the release of cytochrome c and other pro-apoptotic mediators. Together, mitochondrial depolarization, excessive fission, and reduced fusion synergize to accelerate organelle dysfunction, thereby linking mitochondrial failure directly to cellular fate under chronic UVA exposure.

In addition, the chronic accumulation of UVA-induced mitochondrial dysfunction is increasingly recognized as a driver of premature skin aging and degenerative processes, underscoring the importance of targeting mitochondrial protection as a therapeutic strategy. Nutraceuticals with antioxidant and mitochondria-preserving properties have attracted considerable attention for their ability to counteract photooxidative stress [15,16]. Polyphenols have been shown to scavenge ROS [17], stabilize mitochondrial function [18,19], preserve ΔΨm, and restore a healthier balance of fission and fusion events, while activating cytoprotective pathways such as the nuclear factor erythroid 2-related factor 2 (Nrf2)-mediated antioxidant response [19,20], thereby reinforcing endogenous defense mechanisms.

Building on this premise, in our study, we set out to evaluate whether polydatin can ameliorate UVA-induced damage in human dermal fibroblasts. We focused on critical endpoints—cell viability, intracellular ROS levels, mitochondrial functionality (including ΔΨm and dynamics), and activation of Nrf2—to assess whether it exerts cytoprotective effects under photooxidative stress. In doing so, we aim to elucidate whether polydatin functions not only as a direct antioxidant but also as a modulator of mitochondrial integrity, preserving both membrane potential and dynamic remodeling, and as an activator of endogenous defense mechanisms in dermal cells challenged by UVA exposure.

## 2. Materials and Methods

### 2.1. Cells and Treatments

Fibroblasts derived from a healthy control male (CTRL, coded CTRL1) were employed. Fibroblasts were established from a skin biopsy obtained after a signed Informed Consent (prot. N. 9917/15 and prot.cm 10/15 of the Ethics Committee at the Catholic University of Rome). Cell culture was grown in DMEM medium (Sigma Aldrich, St. Louis, MO, USA), supplemented with 10% fetal bovine serum (FBS), 1% penicillin/streptomycin and 2.5% L-glutamine at 37 °C with 5% CO_2_. For the subsequent experiments, cells were seeded at 80% confluency. UVA exposure was produced using a lamp (Vilber Lourmat VL-62C Power 6W; Vilber Lourmat Deutschland GmbH, Eberhardzell, Germany) at 365 nm with a full width at half maximum (FWHM) of ~10 nm, and was placed 10 cm from the source for 2 and 3 h at an intensity of ~0.06 J/cm^2^/s. To minimize radiation uptake by the medium, the cells were kept in PBS during exposure, and immediately after exposure, culture medium was replaced, and the cells were put in an incubator for 24 h before proceeding to the different assays.

Polydatin (purchased from Sigma, St. Louis, MO, USA) was dissolved in DMSO to a final concentration of 10 mM and then, before use, the solution was diluted in PBS to the desired concentrations. Polydatin was added to the cells 24 h before UVA exposure. Additionally, 10 µM polydatin was administered 24 h after UVA exposure. Cells that were not treated with polydatin or exposed to radiation served as controls.

### 2.2. Cell Viability

Cell viability was assessed with the 3-(4,5-dimethylthiazol-2-yl)-5-(3-carboxymethoxyphenyl)-2-(4-sulfophenyl)-2H-tetrazolium (MTS) assay (Promega S.r.l., Padua, Italy), according to the manufacturer’s instructions. Briefly, after UVA exposure and treatment with polydatin, MTS reagent was added to cells, plated in 96-well plates at a cell density of 1 × 10^4^ cells/well. The amount of formazan produced, proportional to the number of live cells, was measured with a plate reader at 490 nm.

### 2.3. ROS Measurement

Intracellular ROS were quantified using the DCF-DA (2′,7′-dichlorofluorescein diacetate) detection kit (Abcam, Cambridge, UK). After the indicated treatments, cells plated in 96-well microplates (50,000 cells/well) were incubated with DCF-DA according to the manufacturer’s instructions. Fluorescence was measured on a CytoFluor Victor3 multi-well plate reader (Victor3-Wallac-1420, PerkinElmer, Waltham, MA, USA). Excitation and emission wavelengths were set to λ_ex = 485 nm and λ_em = 538 nm (bandpass/filter bandwidth as supplied by the reader manufacturer). Fluorescence intensity was expressed as a percentage of the unexposed/untreated control (set to 100%). Technical replicates and the number of independent experiments are reported in Section 2.9.

### 2.4. Nrf2 Detection Assay

Cell lysates were prepared from cultured cells using lysis buffer according to the manufacturer’s instructions. Protein concentrations were quantified using a standard protein assay (n. 23227 Pierce BCA protein Assay kit—Thermo Scientific, Waltham, MA, USA), and equal amounts of total protein were loaded for each sample to ensure comparability across conditions. Nrf2 levels were determined by a sandwich ELISA kit (n. EH348RB, Thermo Scientific), a colorimetric assay, following the protocol provided by the supplier. Absorbance was measured at 450 nm with a microplate reader, and Nrf2 concentrations were calculated based on the standard curve generated with recombinant Nrf2.

### 2.5. Total and Reduced Glutathione Assay

Glutathione levels were measured using a colorimetric assay kit (ab239709, Abcam, Cambridge, UK) to determine total (GSH + GSSG) and reduced glutathione (GSH). Fibroblasts seeded on Petri dishes at 80% confluence were pretreated with 10 µM polydatin for 24 h, followed by exposure to UVA irradiation for 2 to 3 h. After irradiation, the cells were returned to the incubator for 24 h before being lysed using the buffer provided in the kit and centrifuged at 14,000× *g* for 10 min. Protein concentration in the supernatants was evaluated using a BSA calibration curve and a protein assay performed in 96-well microplates (Bio-Rad, Hercules, CA, USA). Glutathione levels were then normalized to the protein content.

The glutathione assay measures absorbance at 412 nm, based on the reaction between glutathione and DTNB (glutathione substrate), forming 2-nitro-5-thiobenzoic acid, a yellow-colored compound. Glutathione reductase was omitted to specifically detect the reduced form of glutathione. The concentrations of total and reduced glutathione were calculated by comparison with the kit-standard respective calibration curves and expressed as micromoles per milliliter (µmol/mL).

### 2.6. Oxygen Consumption Rate

Oxygen consumption rate (OCR) in adherent cells was measured with the Seahorse XF HS Mini Analyzer using the Seahorse XFp Cell Mito Stress Test Kit (Agilent Technologies, Santa Clara, CA, USA). Fibroblasts were seeded in XF HS cell culture microplates at 15,000 cells/well and allowed to grow. The next day, cells were treated with 10 µM polydatin and, after another 24 h, irradiated with UVA for 2 and 3 h. After irradiation, the growth medium was replaced with Agilent Seahorse XF DMEM medium (Agilent, Santa Clara, CA, USA) supplemented with 10 mM glucose, 1 mM pyruvate and 2 mM L-glutamine according to the manufacturer’s protocol, and the cells were incubated at 37 °C for 30 min to allow temperature and pH equilibration. Control cells were unirradiated and untreated cells that were seeded and allowed to grow for 24 h at 37 °C in a 5% CO_2_ humidified incubator and then placed in Seahorse medium. After an OCR baseline measurement, 1 µM oligomycin, 0.2–1.0 µM carbonyl cyanide-p-trifluoromethoxyphenylhydrazone (FCCP), 1 µM rotenone, and 1 µM antimycin A were added sequentially to each well. Prior to each experiment, a titration curve with FCCP was performed to determine the optimal FCCP concentration that maximally stimulated respiration. The acidification rate (ECAR) was simultaneously monitored with OCR under different respiration conditions. All OCR data were normalized to total cellular protein content determined after each experimental run by Bradford assay (Cat. No. B6916, Merck, Darmstadt, Germany).

### 2.7. Mitochondrial Membrane Potential Difference Analysis

To study the difference in mitochondrial membrane potential (ΔΨ), a specific probe was used, JC-9 (Invitrogen, Carlsbad, CA, USA, D22421). This probe is permeable to the mitochondria in a ΔΨ-dependent manner, revealed by a change in fluorescence emission from yellow (~525 nm) to red (~590 nm). Thus, depolarization was indicated by a reduction in the red fluorescence vs. yellow fluorescence ratio. This ratio depends only on ΔΨ and not on other factors (size, form, and density of mitochondria). Cells were seeded in three independent Petri dishes (1 × 10^5^ cells/dish). After 1 h of incubation at 37 °C with 1 µg/mL of JC-9, 50 images were captured and analyzed for each Petri dish. Double acquisition for each image was made: 525 nm for yellow fluorescence and 595 nm for red one. The probe was simultaneously excited by two argon ion lasers at wavelengths of 488 nm and 568 nm, respectively. The ratio between yellow and red fluorescence was calculated pixel by pixel through a software developed by Maulucci and coauthors [21] (http://stke.sciencemag.org/content/1/43/pl3, accessed on 28 August 2025), allowing high-resolution measurement of ΔΨ. For each condition (treated/untreated, exposed/unexposed), 25 images per sample were captured and analyzed. Live-cell confocal imaging was performed using a Nikon A1-MP microscope (Nikon, Tokyo, Japan) equipped with a 60× oil-immersion objective, delivering 80-fs pulses at an 80 MHz repetition rate. Imaging was conducted under controlled environmental conditions (37 °C, 5% CO_2_) provided by an on-stage incubation system (OKOLAB S.r.l., Pozzuoli, Italy). Two fluorescence detection channels were configured: Channel 1 (Ch1) with excitation at 488.3 nm and emission collected through a 525/50 nm bandpass filter, and Channel 2 (Ch2) with excitation at 561.5 nm and emission detected through a 595/50 nm bandpass filter. Images were acquired at a spatial resolution of 2048 × 2048 pixels for both control and treated samples. Images were processed with a custom, ad hoc Python pipeline (NumPy/SciPy/matplotlib). After Gaussian and median denoising and segmentation-based masking, mitochondrial generalized polarization (GP) was computed pixel-wise as:

GP = (I_(595/50) − I_(525/50))/(I_(595/50) + I_(525/50)),

where I_(595/50) and I_(525/50) are the fluorescence intensities recorded in Ch2 (561.5 nm excitation; 595/50 nm emission) and Ch1 (488.3 nm excitation; 525/50 nm emission), respectively. Per-image GP means were then used for group summaries.

### 2.8. Fis1 and Mitofusin2 Levels Detection

The proteins human Fission 1 (FIS1) and human Mitofusin 2 (MFN2) were determined in cell lysates, processed according to experimental protocol, using ELISA kits following the manufacturer’s protocols (MNF2 cat. MBS2024886 by Mybiosource and FIS1 cat MBS453299 by Mybiosurce Inc., P.O. Box 153308: San Diego, CA 92195-3308: USA). Briefly, after determining standard curves, samples of unknown concentration (50 µL of 0.5 mg protein/mL of cell lysate) were added, in triplicate, to wells pre-coated with antibodies. The plate, after addition of the antibody cocktail, was read at 450 nm with a microplate reader. The concentration of FIS1 and MFN2 in the samples was calculated in ng/mL by interpolating the absorbance values of the standard curve and expressed as a percentage value compared with untreated and unexposed control cells.

### 2.9. Statistical Analysis

Each experiment was replicated at least three times and up to eight replicates per group. Results are displayed as means ± SEM. Data were analyzed by one-way ANOVA using GraphPad Prism ver. 8 software (San Diego, CA, USA). The level of significance was set at *p* ≤ 0.05. For the JC-9 generalized polarization imaging of mitochondrial ΔΨm, group differences were assessed using a Python code by one-way ANOVA (with Levene’s test and a Kruskal–Wallis robustness check), followed by post hoc pairwise Welch *t*-tests with adjustment for multiple comparisons (*p* ≤ 0.05).

## 3. Results

**Figure 2:**
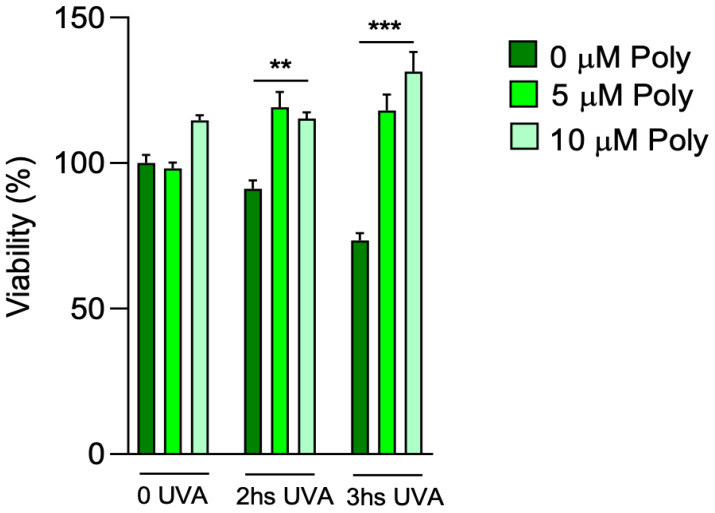
Cell viability in fibroblasts exposed to UVA irradiation pre-treated with 5 and 10 μM of polydatin. Cell viability was expressed as % of the control (unexposed and untreated cells). Statistical analysis was performed by comparing data under the same experimental conditions (0, 2 and 3 h UVA exposure). Note the cytoprotective effect of polydatin after 2 and 3 h of UVA exposure. Bars represent mean + SD. ** *p* < 0.01, *** *p* < 0.001 (between untreated cells, 0 µM) by one-way ANOVA (with Geisser-Greenhouse’s correction).

We first tested the response of fibroblasts exposed to different times of UVA irradiation by evaluating their viability. Based on our previous results [22], a concentration titration curve was performed at 5 and 10 µM of polydatin to determine the better concentration of pre-treated compound, followed by 2 and 3 h of UVA exposure (Figure 2). After UVA exposure, untreated fibroblasts showed a loss of viability of 10 and approximately 30% after 2 and 3 h, respectively, compared to control cells that were not exposed to UVA but kept under a hood outside the incubator. Pretreatment with 5 and 10 µM of polydatin had no effect on cell viability in unexposed fibroblasts. Polydatin protected cells from UVA-dependent cytotoxicity even at low concentrations (5 µM). This effect was even more pronounced at 10 µM of the compound, with increases in cell viability after 3 h of UVA exposure. Thus, 10 µM polydatin was chosen for the following experiments.

**Figure 3:**
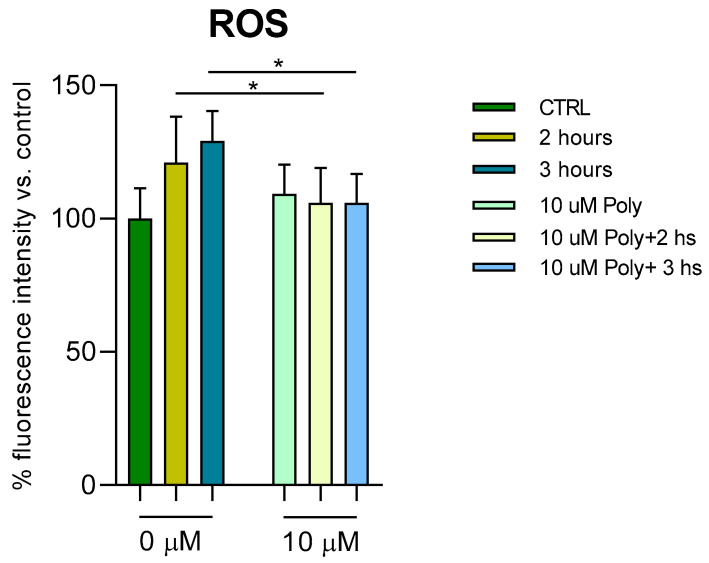
Reactive oxygen species (ROS) for fibroblasts unexposed (in green) and exposed to UVA for 2 h yellow bars) and 3 h (heavenly bars) in the absence (0 µM) and pre-treated with 10 µM of polydatin. ROS were expressed as a percentage of fluorescence intensity compared with the unexposed and untreated control (CTRL), arbitrarily considered 100%. Statistical analysis was performed by comparing data between unexposed vs. exposed and between untreated vs. treated fibroblasts. Bars represent mean + SD. * *p* < 0.05 (between corresponding controls) by one-way ANOVA (with Geisser-Greenhouse’s correction).

To study the molecular mechanisms underlying UVA damage and the protective effect of polydatin, we measured the presence of ROS after administration of 10 µM polydatin before UVA exposure for 2 and 3 h, respectively (Figure 3). ROS were measured as fluorescence intensity and expressed as a percentage compared with unirradiated, untreated cells (arbitrarily set as 100%). In treated cells, an increase of around 10% in ROS after 2 and 3 h of UVA exposure is observed. In the polydatin-pretreated cells, the oxidation levels return to normal levels.

**Figure 4:**
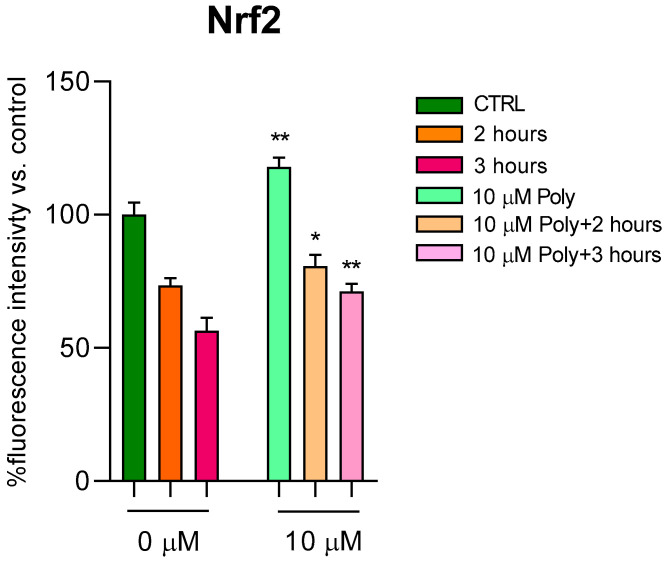
Nrf2 dosage after 2 and 3 h of UVA irradiation with and without 10 µM of polydatin. Results are presented as a percentage of protein compared with untreated and unexposed control (arbitrarily set as 100%, light green bar). Polidatyn induced an increase in Nrf2 levels in unexposed, while in exposed (2 and 3 h) and treated fibroblasts, a slight increase in its levels is observed. Bars express mean + SD. * *p* < 0.05, ** *p* < 0.01 (between untreated cells) by one-way ANOVA (with Geisser-Greenhouse’s correction).

Since the direct correlation between ROS and Nrf2 levels is known, which seeks to counteract cellular oxidation, we went on to dose this protein in fibroblasts treated according to the previous experimental protocol. In Figure 4, we reported Nrf2 values in cells unexposed and in cells exposed for 2 and 3 h to UVA irradiation, with and without pre-treatment with 10 µM of polydatin. Even in cells not exposed to radiation, polydatin evidently resulted in a significant increase in Nrf2 levels, and, after UVA exposure, an increase at 2 and 3 h of radiation exposure is observed.

Since Nrf2 is known to enhance cellular antioxidant defenses through multiple mechanisms, including the increase of overall antioxidant potential [23,24], we evaluated cytosolic glutathione content in treated fibroblasts according to our experimental design (see Table 1).

Under basal conditions, Polydatin treatment did not significantly alter either total glutathione (GSSG + GSH: 0.241 ± 0.065 µmol/mL vs. 0.244 ± 0.042 µmol/mL in control) or reduced glutathione (GSH: 0.173 ± 0.065 µmol/mL vs. 0.195 ± 0.033 µmol/mL in control), indicating that the compound does not affect the physiological redox balance of fibroblasts.

After 2 h of UVA exposure, reduced glutathione was significantly decreased (0.098 ± 0.049 µmol/mL), while total glutathione levels remained essentially unchanged (0.257 ± 0.049 µmol/mL), suggesting that UVA irradiation primarily reduces the GSH form, likely via oxidative conversion to GSSG. Similarly, after 3 h of UVA exposure, GSH was further reduced (0.093 ± 0.033 µmol/mL), whereas total glutathione remained largely constant (0.247 ± 0.049 µmol/mL). These data indicate that fibroblasts consume reduced glutathione to counteract the ROS increase induced by UVA.

Pretreatment with Polydatin significantly preserved GSH levels in UVA-exposed fibroblasts at both 2 h (0.195 * ± 0.098 µmol/mL vs. 0.098 ± 0.049 µmol/mL without Polydatin) and 3 h (0.128 * ± 0.065 µmol/mL vs. 0.093 ± 0.033 µmol/mL without Polydatin). Total glutathione levels did not show significant changes in any of the UVA + Polydatin conditions (2 h: 0.251 ± 0.075 µmol/mL; 3 h: 0.228 ± 0.064 µmol/mL), indicating that Polydatin specifically preserves the reduced form without affecting the total pool.

Overall, these findings demonstrate that Polydatin exerts a protective antioxidant effect under UVA-induced oxidative stress by maintaining intracellular GSH levels.

**Figure 5:**
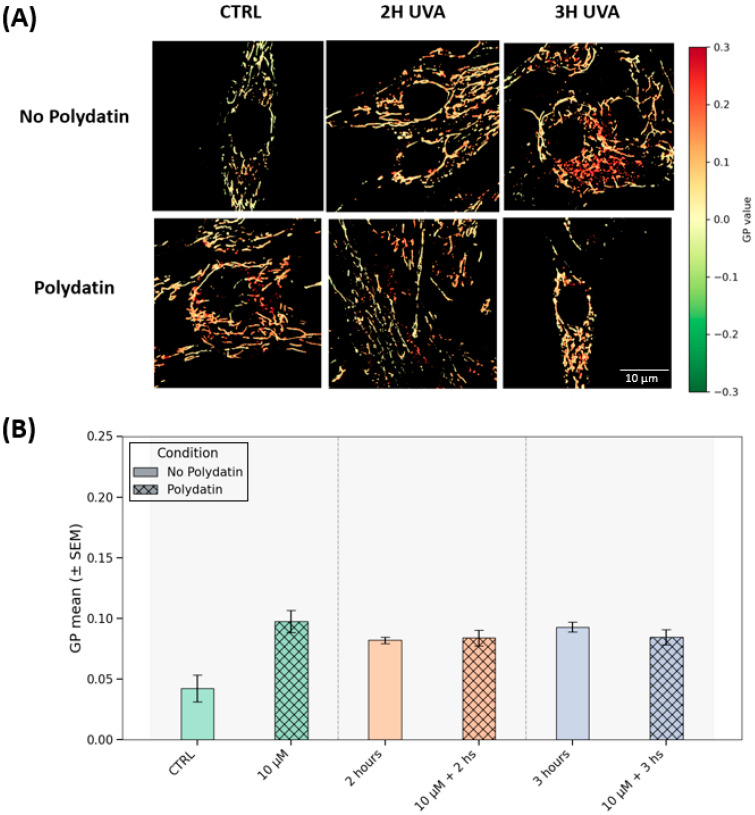
ΔΨm by generalized polarization (GP). (**A**) Representative GP maps for six conditions: columns show CTRL, 2 h UVA, and 3 h UVA; rows show untreated (“No Polydatin”) and polydatin-treated cells. Although GP is defined on [–1, 1], the display range is intentionally restricted (see color bar) to enhance visual contrast across conditions. Scale bar, 10 µm. (**B**) Quantification of GP for the same groups. Bars show group means ± SEM. Solid bars denote untreated samples; cross-hatched (diamond) bars denote treated samples.

Mitochondrial generalized polarization (GP) analysis indicates that the mitochondrial membrane potential (ΔΨm) is stable across UVA exposure and polydatin treatment. In Figure 5A, GP maps are qualitatively similar across all UVA conditions (2 h and 3 h, with or without polydatin) and in the treated control. Figure 5B consolidates the quantification, with values tightly clustered (~0.08–0.010) for those groups, with a lower trend in the untreated control. Following the statistical analysis and post hoc between-group comparisons, no contrasts reached significance (adjusted p for multiple comparisons = 0.08–1.00). Within this experimental window, UVA (2–3 h) did not lead to a detectable depolarization at the endpoint, and polydatin did not measurably shift ΔΨm within the sensitivity of our assay; the isolated decrease in the untreated control is most consistent with baseline variability. In keeping with the ROS readouts (attenuated oxidative burden under polydatin), any protective effect may primarily reflect redox modulation rather than a pronounced alteration of mitochondrial bioenergetic status.

**Figure 6:**
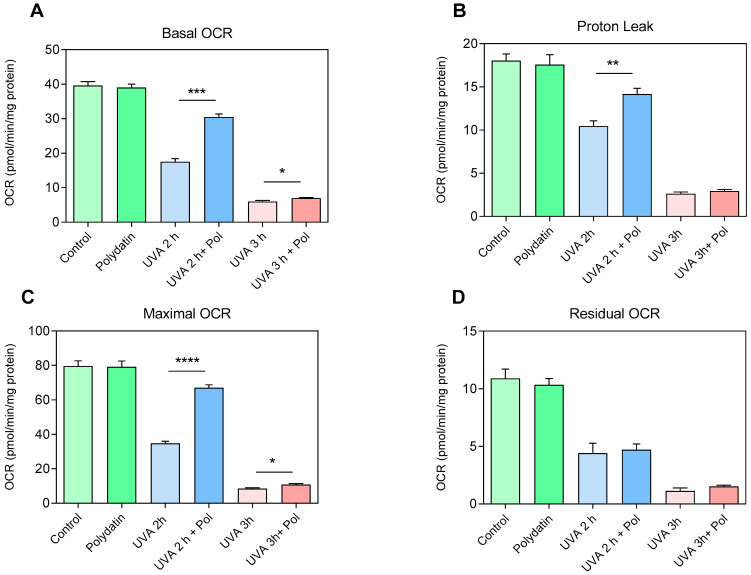
Oxygen consumption rate (OCR) of fibroblasts after 2 h and 3 h of UVA exposure with and without 10 µM of Polydatin. (**A**) Basal oxygen consumption rate (Basal OCR), (**B**) Proton Leak, (**C**) Maximal OCR, and (**D**) non-mitochondrial respiration (Residual OCR) were expressed as (pmol/min/mg protein) and are average values ± SEM of three independent experiments performed in duplicate. * *p* < 0.05; ** *p* < 0.01; *** *p* < 0.001; **** *p* < 0.0001 (between corresponding controls) by one-way ANOVA (with Geisser-Greenhouse’s correction).

To assess the protective effects of polydatin on the respiratory capacity of human fibroblasts, examination of metabolic activity using the Seahorse XF Cell Mito Stress Assay was performed on cells exposed to UVA radiation (2 and 3 h) with and without pre-treatment with 10 μM of polydatin (Figure 6). Regarding basal respiration (Basal OCR, Figure 6A), cells treated with polydatin alone did not display significant changes compared to controls, indicating that the compound does not affect mitochondrial function under physiological conditions. In contrast, UVA exposure induced a marked reduction in oxygen consumption after 2 h, which became more pronounced after 3 h. Co-administration of polydatin partially preserved basal respiration at 2 h but was ineffective at 3 h, suggesting a time-limited protective effect. The proton leak (Figure 6B) exhibited a similar pattern. In untreated fibroblasts, UVA exposure progressively reduced the oxygen consumption linked to dissipation of the proton gradient, indicative of inner mitochondrial membrane damage. Polydatin attenuated this reduction after 2 h, while no significant protection was observed at 3 h. Analysis of maximal respiratory capacity (Maximal OCR, Figure 6C) confirmed a severe functional deficit induced by UVA exposure: fibroblasts exposed for 2 h already exhibited a significant reduction in spare respiratory capacity, which was almost completely abolished after 3 h. The presence of polydatin partially restored maximal capacity at 2 h; the residual OCR (Figure 6D), reflecting non-mitochondrial oxidative processes, did not show substantial differences among experimental conditions. This suggests that UVA-induced damage, as well as the protective action of polydatin, are specifically directed toward mitochondrial functionality, without significantly affecting extra-mitochondrial oxidative processes. Overall, the data indicate that 10 µM polydatin exerts a protective effect against UVA-induced oxidative stress, partially preserving both basal respiration and maximal respiratory capacity. However, this protection is evident only after 2 h of exposure and is lost at 3 h, highlighting a limit to the compound’s ability to counteract mitochondrial damage under severe stress.

**Figure 7:**
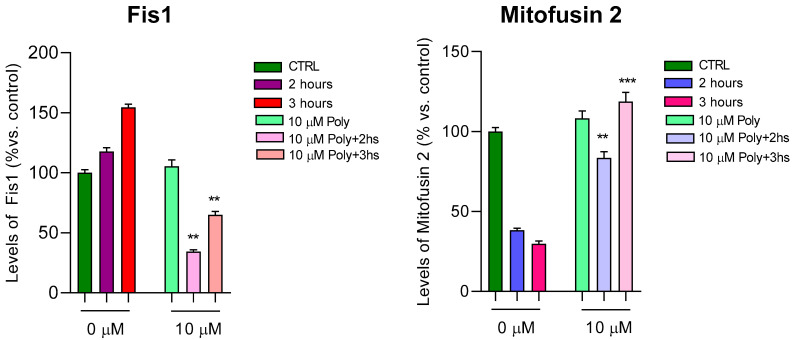
Fis1 and Mitofusin2 dosage after 2 and 3 h of UVA irradiation with and without 10 µM of polydatin. Results are presented as a percentage of proteins compared with untreated and unexposed control (arbitrarily set as 100%). UVA irradiation induced an increase in Fis1 levels (panel on the left) and a complementary reduction in MFN2 levels (panel on the right). Fis1 and MFN2 levels tend to normalize pre-treating cells with polydatin, indicating its protective effects against mitochondrial damage. Bars express mean + SD. ** *p* < 0.01; *** *p* < 0.001 (between untreated cells) by one-way ANOVA (with Geisser-Greenhouse’s correction).

Fusion and fission activity of mitochondria was assessed through an ELISA assay dosing levels of Mitofusin2 (MFN2), a mitochondrial membrane protein that regulates mitochondrial fusion, and Fis1, which is a component of the mitochondrial complex, ARCosome, that promotes mitochondrial fission. Dosage of both proteins was detected after 2 and 3 h of UVA irradiation of fibroblasts pre-treated with polydatin, as shown in Figure 7. Fis1 showed an increase after UVA irradiation, as expected, indicating mitochondrial damage, particularly after 3 h of rays. Its levels were reduced after pre-treatment of cells with polydatin, demonstrating a protective effect against UVA rays. Similarly, levels of Mitofusin2 decreased after UVA irradiation, corresponding to low levels of mitochondrial activity associated with fusion of these organelles. Pre-treatment with polydatin and then irradiating cells led to an increase in Mitofusin2 levels towards those of the control, highlighting again the protective effect of the compound against the induced damage.

## 4. Discussion

This study demonstrates that polydatin provides effective protection against UVA-induced damage in human dermal fibroblasts by preserving mitochondrial integrity, reducing oxidative stress, and modulating antioxidant defense pathways. Pre-treatment with polydatin significantly improves cell viability after UVA exposure, reduces intracellular ROS, and regulates proteins involved in mitochondrial dynamics and the antioxidant response. UVA radiation is well known to cause oxidative stress primarily through increased ROS production, leading to mitochondrial dysfunction, impaired bioenergetics, and cell death in dermal fibroblasts [25]. The observed elevation of ROS following UVA exposure aligns with previous reports, where oxidative stress mediates photoaging and dermal damage [9]. Polydatin effectively reduces ROS levels toward baseline after UVA irradiation, confirming its potent antioxidant activity. This action is attributed to its polyphenolic structure, which facilitates direct ROS scavenging and interrupts oxidative chain reactions [4].

Mitochondrial dysfunction is a key feature of UVA-induced cellular injury, characterized by loss of bioenergetic capacity, increased mitochondrial fission, and decreased fusion resulting in fragmented and dysfunctional mitochondria [10,26]. Within the sensitivity of our assay, Δψm did not show significant alterations after UVA exposure; however, the overall trend, together with preserved mitochondrial respiration and dynamics, suggests that polydatin helps sustain mitochondrial bioenergetics.

This interpretation is supported by the normalization of mitochondrial dynamic regulators: polydatin prevented the UVA-induced increase in the fission protein Fis1 and the decrease in the fusion mediator Mitofusin-2 (MFN2), thus maintaining mitochondrial balance close to physiological conditions. Regulation of mitochondrial dynamics is a critical target for preventing photoaging [26].

A novel finding emerging from our Seahorse metabolic analysis indicates that polydatin pre-treatment preserves mitochondrial respiration in UVA-exposed fibroblasts. Maintenance of basal and maximal respiration rates suggests that polydatin sustains mitochondrial oxidative phosphorylation capacity despite oxidative insult. Preserved mitochondrial respiration is essential for ATP production and cell survival, preventing energy depletion-induced cell death pathways triggered by UVA damage [27]. This functional mitochondrial protection expands the mechanistic understanding beyond preservation of Δψm, confirming polydatin’s role in maintaining mitochondrial bioenergetics and energy capacity, which are crucial for cellular homeostasis [20]. Furthermore, our data show that polydatin did not directly up-regulate Nrf2 expression but rather prevented the UVA-induced suppression of Nrf2 expression and nuclear translocation. Through this mechanism, polydatin preserved Nrf2-mediated antioxidant responses, leading to enhanced redox homeostasis and elevated reduced glutathione (GSH) levels [19]. Importantly, polydatin administered after UVA exposure failed to rescue cell viability, indicating that its protective activity is predominantly preventive and relies on the pre-activation of antioxidant and mitochondrial defense systems.

Notably, recent research by Valenti et al. [16] provides additional insights into polydatin’s mitochondrial protective effects, demonstrating that it reactivates mitochondrial bioenergetics and mitophagy while preventing premature senescence through modulation of microRNA-155 and its downstream targets in human dermal fibroblasts derived from a donor with trisomy 21 (Down syndrome). This evidence supports the broad potential of polydatin in mitigating mitochondrial dysfunction and cellular aging processes beyond photooxidative stress.

Recent studies [28] have also helped to identify the primary molecular targets of UVA irradiation in skin cells, providing a mechanistic context for our findings. UVA photons (320–400 nm) penetrate deeply into the dermis and are absorbed by several endogenous chromophores, including flavins, porphyrins, NADH/NADPH-dependent enzymes, and cytochrome oxidases, leading to the generation of reactive oxygen species (ROS) such as singlet oxygen, superoxide anion, and hydrogen peroxide. These ROS act as secondary messengers that modulate redox-sensitive signaling pathways, particularly those involving MAPKs, NF-κB, and Nrf2, thereby influencing gene expression, mitochondrial function, and cell survival. The oxidation of mitochondrial and cytosolic targets alters calcium homeostasis, depolarizes the mitochondrial membrane potential, and triggers adaptive antioxidant responses. Moreover, protein oxidation and lipid peroxidation caused by UVA exposure contribute to extracellular matrix disorganization and premature cellular senescence. Overall, these mechanisms underline how UVA light exerts its biological effects mainly through photooxidative damage to redox-active chromophores and subsequent signaling dysregulation, rather than through direct DNA absorption, which is more typical of UVB radiation [29,30].

In addition to our findings, several natural polyphenols have been reported to exert protective effects against UVA-induced skin damage, mainly through antioxidant activity and modulation of inflammatory responses [31,32]. The regulation of mitochondrial dynamics has recently emerged as a crucial node in photoaging processes, since imbalances in fusion and fission directly contribute to bioenergetic dysfunction and premature senescence [33,34]. In this context, the involvement of Nrf2 provides an additional layer of protection, as its activation by natural compounds reduces oxidative burden and reinforces endogenous antioxidant capacity [35,36]. Moreover, other plant-derived molecules, such as epigallocatechin gallate, have shown effects comparable to those described here for polydatin, preserving mitochondrial integrity and reducing oxidative stress in skin fibroblasts [36]. These findings strengthen the translational relevance of polydatin and situate our results within a broader framework of nutraceutical strategies aimed at counteracting photooxidative stress and skin photoaging.

## 5. Conclusions

In conclusion, polydatin exerts multifaceted protective effects in human dermal fibroblasts exposed to UVA by preserving mitochondrial function and respiration, modulating mitochondrial dynamics, reducing ROS accumulation, and activating the Nrf2-mediated antioxidant response. These findings underscore polydatin’s promising potential as a therapeutic agent for preventing photoaging and oxidative skin damage and warrant further in vivo and clinical investigations.

## Figures and Tables

**Table 1:** µM polydatin, and unexposed or exposed to UVA irradiation for 2 or 3 h. Total and reduced glutathione concentrations are expressed per µmol/mL. Values were calculated as described in Section 2.

Condition	GSSG + GSH (µmol/mL)	GSH (µmol/mL)	Statistical Significance
Control	0.244 ± 0.042	0.195 ± 0.033	ns (Total)
Control + Polydatin	0.241 ± 0.065	0.173 ± 0.065	ns (Reduced GSH)
UVA 2 h	0.257 ± 0.049	0.098 ± 0.049	ns (Total)
UVA 2 h + Polydatin	0.251 ± 0.075	0.195 * ± 0.098	* ↑ (Reduced GSH)
UVA 3 h	0.247 ± 0.049	0.093 ± 0.033	ns (Total)
UVA 3 h + Polydatin	0.228 ± 0.064	0.128 * ± 0.065	* ↑ (Reduced GSH)

Statistical significance was assessed by one-way ANOVA, comparing each treated group with its corresponding untreated control (without polydatin). ns = not significant (*p* ≥ 0.05); * *p* < 0.05, treated vs. corresponding untreated control. The arrow indicates a significant increase.

## Data Availability

All data supporting the findings of this study are available within the article.
